# The efficient plant genetic transformation system mediated by FELBs and RTBs respectively *via* novel somatic embryogenesis in *Allium sativum*

**DOI:** 10.3389/fpls.2026.1832239

**Published:** 2026-07-06

**Authors:** Kedong Xu, Xinliang Shao, Ruifang Bu, Rongxin Ji, Mengqi Lu, Yulu Shi, Fangzhi Tao, Nan Wu, Siyu An, Yawen Zhao, Yaxu Ping, Yingqian Yan, Shen Fu, Jiaxin Xi, Bingyan Song, Jian Wang, Chengwei Li

**Affiliations:** 1 Key Laboratory of Plant Genetics and Molecular Breeding, Henan Key Laboratory of Crop Molecular Breeding and Bioreactor, Henan International Joint Laboratory of Translational Biology, Zhoukou Normal University, Zhoukou, China; 2 Henan Plant Gene and Molecular Breeding Engineering Research Center, Zhoukou Normal University, Zhoukou, China; 3 College of Life Science, Henan Agricultural University, Zhengzhou, China; 4 Wheat Research Institute, Zhoukou Academy of Agricultural Sciences, Zhoukou, China; 5 College of Agriculture, Zhengzhou University, Zhengzhou, China

**Keywords:** *Allium sativum*, frog egg-like body, plant growth regulators, rhizoid tuber, somatic embryogenesis

## Abstract

**Introduction:**

Somatic embryogenesis represents an indirect but highly efficient biotechnological method for plant regeneration, genetic transformation, and virus-free propagation.

**Methods:**

Herein, we investigated the effects of NAA, 2,4-D, and TDZ on the morphogenic capacity of *Allium sativum*. This is evaluated in terms of the formation of frog egg-like body and rhizoid tuber morphological structures, somatic embryo development, shoot regeneration, and the genetic transformation method.

**Results:**

Two novel high-efficiency SE systems in garlic were characterized, operating through the FELB and RTB pathways, respectively, with a focus on the regulatory effects of plant growth regulators (PGRs) on SE progression. In the FELB pathway, somatic embryos were highly induced from root explants, achieving an induction rate of 96.33%. Embryoid development was successfully accomplished using a combination of 2.5 mg/L NAA and 2.5 mg/L 2,4-D under dark conditions. Furthermore, 5.0 mg/L BAP was identified as the optimal cytokinin concentration for plant regeneration *via* the FELB pathway. In the RTB pathway, somatic embryos were also efficiently induced from root explants; under sucrose semistarvation conditions, the average number of RTBs induced per explant reached 44.067. Embryoid development in the RTB pathway was achieved with 10 mg/L NAA under dark conditions combined with 20.0 mg/L TDZ under light conditions (180 µmol·m^-2^s^-1^), a combination that significantly promoted RTB formation. Isolated RTBs were successfully germinated on MS medium supplemented with 2.5 mg/L BAP. This study is the first to reveal that the developmental stages and ontogenetic processes of FELBs and RTBs in the monocotyledonous plant garlic exhibit striking homology to those reported in dicotyledonous species. Using *RcLEC1-B* as a reporter gene, we confirmed the successful establishment of FELB/RTB-mediated genetic transformation; the *TCS* gene was further introduced to enhance garlic resistance against *Penicillium chrysogenum* and *Phytophthora porri*.

**Discussion:**

Collectively, our findings establish novel methodologies for garlic SE and transgenic manipulation, providing valuable tools for the genetic improvement of this economically important crop.

## 1 Introduction

*Allium* comprises nearly 500 species with high edible, ornamental, and medicinal values (Wai-Jo et al., 2020; Asgharpour et al., 2021; Forma et al., 2021; Hu et al., 2022; Vezza et al., 2024) and possesses multiple health-promoting pharmacological properties. As an economically important crop within the genus *Allium*, *Allium sativum* (garlic) is cultivated globally. It contains a diverse range of bioactive compounds, including sulfur-containing compounds such as allicin and vinyl disulfide, as well as flavonoids such as quercetin. These metabolites confer broad-spectrum biofunctions including anticancer effects (Rauf et al., 2022), anti-inflammatory properties (Santos-Álvarez et al., 2024), antioxidant capacity, antidiabetic activity (Jiang et al., 2024), kidney-protecting function, anti-atherosclerotic action, antifungal and antiviral activities, as well as antihypertensive effects (El-Saber Batiha et al., 2020).

Garlic cultivation area has continuously expanded over recent decades. However, garlic is obligately sterile and exclusively propagated *via* vegetative bulbs. This inherent reproductive constraint results in low propagation efficiency, progressive accumulation of phytopathogens, and frequent outbreaks of viral, fungal, and bacterial diseases during commercial production (Pappu et al., 2005; Chodorska et al., 2014), which substantially limit garlic yield, quality stability, and germplasm renewal. Accordingly, breeding disease-resistant garlic cultivars has become an urgent requirement for industrial sustainable development. Several wild *Allium* species, including *A. chinense*, *A. macrostemon*, *A. roborowskianum* and *A. prattii*, etc. harbor abundant stress-resistant genes and provide elite genetic resources for garlic molecular improvement. Nevertheless, efficient genetic engineering platforms for garlic are still lacking.

Genetic transformation and molecular breeding are pivotal strategies to overcome production bottlenecks and achieve durable disease resistance in garlic. However, establishing a high-efficiency plant regeneration and genetic transformation system still represents a major technical constraint. Since the 1970s, numerous studies have focused on direct organogenesis in garlic using diverse explants, such as young leaves (Havránek and Novák, 1973), root apices (Shuto et al., 1993; Shahidul Haque et al., 1997; Barandiaran et al., 1999), shoot tips (Nagakubo et al., 1993), protoplasts derived from shoot primordia (Ayabe et al., 1995), stem discs (Ayabe and Sumi, 1998), and suspension cultures of inner fleshy scale leaves of bulbs containing basal disc regions (Madhumita et al., 2005). Meanwhile, research on indirect regeneration has mainly focused on organogenesis and somatic embryogenesis from callus induced from garlic stem tips, bulb leaf discs, and clove stem segments (Mostafa, 1977). Early attempts at garlic genetic transformation predominantly adopted particle bombardment, which suffered from limited receptor materials and poor experimental reproducibility. Tested explants included callus, leaves, and cloves (Park et al., 2002), immature bulbs (Barandiaran et al., 1998), embryogenic callus and basal plate discs (Ferrer et al., 2000), callus derived from apical and non-apical root segments (Ahn et al., 2013), true garlic seeds, and bulbils (Zheng et al., 2004). Although conventional somatic embryogenesis has been reported in *Allium*, existing systems generally show low regeneration competence and unstable transformation efficiency. Furthermore, other explants, such as shoot primordium-like tissues derived from apical meristems (Kondo et al., 2000), immature embryos (Eady et al., 2005), and immature leaves (Kenel et al., 2010), have also been used as receptor materials for genetic modification in garlic. Due to the low proliferation rate, limited genetic improvement potential, and continuous germplasm degradation in garlic, a stable and reproducible transformation system based on somatic embryogenesis has not yet been fully established. Such a system is expected to provide an efficient and promising approach for mass clonal propagation and agronomic trait modification in *Allium* crops.

Somatic embryogenesis is widely recognized as an ideal technical platform combining virus elimination and high-frequency plant regeneration, and also serves as an excellent receptor system for genetic transformation (Pathi et al., 2013; Xu et al., 2019b). The main morphotypes derived from somatic embryogenesis can be categorized into two groups: 1) Classic somatic embryo (SE) structures. In dicots, somatic embryos develop sequentially from pro-embryos to globular, heart-shaped, torpedo-shaped, and cotyledonary stages. In monocots, the developmental pathway proceeds through pro-embryos, globular embryos, and pear- or shield-shaped embryos. In gymnosperms, embryos progress from early to late developmental stages and ultimately form mature cotyledonary embryos (Smertenko and Bozhkov, 2014). In pteridophytes, somatic embryos may develop into linear pro-embryos and subsequent early and late embryonic leaves (Mikuła et al., 2015), or form direct and indirect green globular bodies (GGBs) (Li et al., 2015). 2) Protocorm-like bodies (PLBs), which have been extensively documented in Orchidaceae (Fang et al., 2016), Araceae (Gantait et al., 2012), Liliaceae (Nhut et al., 2002), and Rosaceae (Tian et al., 2008; Liu et al., 2014).

Notably, our previous studies identified two novel embryogenic structures, namely frog egg-like bodies (FELBs) (Xu et al., 2014, Xu et al., 2016) and rhizoid tubers (RTBs) (Xu et al., 2015). Although classic SE structures have been described in *Allium* species (Lu et al., 1989; Kim and Soh, 1996; Sata et al., 2000; Malik et al., 2020), FELBs and RTBs differ markedly from conventional somatic embryos in morphological characteristics, developmental progression, and proliferation capacity, thereby possessing unique research and application values. The physical separation of embryonic units from maternal tissues facilitates virus elimination in regenerated plantlets. Their stage-dependent developmental features are highly compatible with *Agrobacterium*-mediated genetic transformation procedures. In addition, the organic aggregation of multiple embryonic units greatly improves the proliferation efficiency of regenerated and transgenic plantlets. Notably, to date, FELBs and RTBs have only been described in a small number of dicotyledonous species (Xu et al., 2014, Xu et al., 2015, Xu et al., 2016; Saeed et al., 2019), with no relevant reports in monocotyledons.

Against this background, the present study established an efficient plant regeneration and genetic transformation system in garlic *via* FELB- and RTB-mediated somatic embryogenesis. This established system provides a robust technical foundation for rapid clonal propagation, virus eradication, functional validation of exogenous genes, and innovative breeding of disease-resistant garlic germplasm. Future research will focus on dissecting the molecular regulatory networks governing FELB and RTB formation and development, and screening key regulators associated with plant growth regulators (PGRs) and environmental conditions, so as to further optimize this embryonic regeneration and transformation system.

## 2 Materials and methods

### 2.1 Plant materials

**Figure 1:**
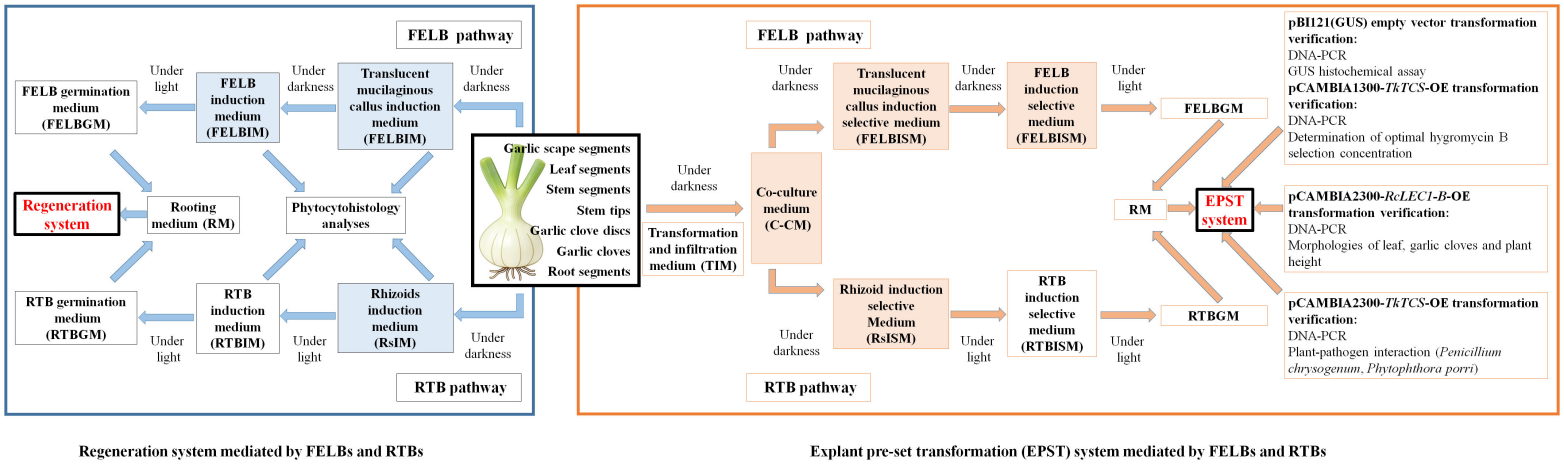
The experimental workflow of somatic embryogenesis and genetic transformation in garlic mediated by FELBs and RTBs.

In this study, the cultivated garlic “Dinghongzao”, a medium-ripening local variety widely grown in China, was used as the experimental material. We aimed to establish FELB- and RTB-mediated somatic embryogenesis systems, as well as the corresponding genetic transformation systems in garlic, and further validate the applicability of these two embryogenic regeneration pathways. The detailed experimental workflow is illustrated in **Figure 1**. Garlic clove explants were surface-sterilized with 75% (v/v) ethanol for 1–3 min, followed by treatment with 0.1% (m/v) mercuric chloride for 9–10 min. Thereafter, the explants were rinsed 3–5 times with sterile distilled water. After disinfection, the explants were inoculated onto half-strength MS medium (1/2 MS; Murashige and Skoog, 1962) supplemented with 10 g/L sucrose and 6.5 g/L agar (pH 5.8) for root induction and sterile seedling establishment. Cultures were maintained at 25 ± 1 °C under a 16 h light/8 h dark photoperiod, with a light intensity of 150 µmol·m^-2^s^-1^.

### 2.2 Preparation and inoculation of explants

In this study, multiple explant types were used for the induction of FELBs and RTBs, as described below: root segments (approximately 1–2 cm in length) and shoot tips (bearing one leaf primordium, roughly 1 cm long), excised from sterile seedlings; garlic cloves (removed of root primordia and dormant buds) and garlic clove discs (about 1–2 mm thick), which were surface-sterilized following the aforementioned protocol; stem segments, leaf segments, and garlic scape segments (roughly 1–2 cm in length), collected from potted garlic seedlings.

### 2.3 One-step induction of FELB and generation of FELBs

To optimize plant growth regulator (PGR) conditions for the induction of translucent mucilaginous callus and FELBs, two auxin analogs were tested via three experimental schemes using FELB induction medium (FELBIM). The basal medium consisted of MS salts and MS vitamins, supplemented with 30.0 g/L sucrose and 3.45 g/L gellan gum. Four concentrations (0, 1.25, 2.5, and 3.75 mg/L) of 1-naphthaleneacetic acid (NAA) or 2,4-dichlorophenoxyacetic acid (2,4-D) were applied either individually or in combined NAA and 2,4-D treatments. All explants were cultured on FELBIM and incubated in darkness at 25 ± 1 °C to induce the formation of translucent mucilaginous callus and FELBs.

FELBs at the heart-to-torpedo transitional and torpedo embryonic stages were collected, rinsed with sterile distilled water to remove surface translucent mucilaginous callus, and then transferred to FELB germination medium (FELBGM). FELBGM contained MS salts, MS vitamins, 30.0 g/L sucrose, 6.5 g/L agar, and 5.0 mg/L 6-benzylaminopurine (BAP), with the pH adjusted to 5.8. Cultures were maintained at 25 ± 1 °C under a 16 h light/8 h dark photoperiod at a light intensity of 180 µmol·m^-2^·s^-1^ to promote FELB germination and plantlet regeneration; subculturing was performed at monthly intervals. When plantlets reached 2–3 cm in height, they were separated and transferred to rooting medium (RM). RM was prepared with half-strength MS salts and half-strength MS vitamins, and supplemented with 10 g/L sucrose and 0.15 mg/L 3-indolebutyric acid (IBA).

### 2.4 Two-step induction of rhizoids and generation of RTBs

All garlic explants were cultured on rhizoid induction medium (RsIM), which was supplemented with MS salts, MS vitamins, 3.45 g/L gellan gum, 2,4-D and/or NAA at gradient concentrations (0, 5.0, 10.0, and 15.0 mg/L), and sucrose at concentrations of 0, 7.5, 15.0, 22.5, and 30.0 g/L (pH 5.8). The explants were incubated in the dark at 25 ± 1°C to induce rhizoid formation. For RTB induction, the rhizoid clusters, along with their original explants, were *in situ* transplanted onto RTB induction medium (RTBIM). This medium consisted of MS salts, MS vitamins, 3.45 g/L gellan gum, and thidiazuron (TDZ) at concentrations of 0, 10.0, 20.0, and 30.0 mg/L (pH 5.8), and the cultures were maintained under high light intensity (180 µmol·m^-2^·s^-1^).

Isolated RTBs were germinated on RTB germination medium (RTBGM), which was an MS medium supplemented with 2.5 mg/L BAP. The culture conditions, including light intensity, photoperiod, temperature, and subculture schedule, were consistent with those described above. During the embryoid germination process, the embryos growing outside the RTB structures could also be *in situ* regenerated on the original parent tissue. The rooting induction method for RTB-derived regenerated plantlets was identical to that for FELB-derived plantlets.

### 2.5 Phytocytohistology analyses of the developmental process of FELBs, rhizoids and RTBs

FELBs, rhizoids, and RTBs at different developmental stages were fixed in 4% paraformaldehyde (dissolved in 100 mM phosphate buffer solution [PBS], pH 7.2) for 36–48 hours. Subsequently, the samples were sequentially dehydrated in 10% and 20% sucrose solutions, with each dehydration step lasting 20–24 hours. They were then embedded in optimum cutting temperature (OCT) compound for 2–3 hours.

The embedded samples were sectioned into 5-8 μm thick slices using a cryostat microtome (CM1850, Leica Microsystems, Germany) and observed under an Olympus BX43 microscope, following the methods described by Xu et al. (2014, Xu et al., 2015, Xu et al., 2016) and Delgado-Aceves et al. (2025).

### 2.6 Screening of representative reference genes for genetic transformation identification

The binary overexpression vector pBI121 (GenBank accession no.: AF485783.1), which contains the *β-glucuronidase* (*GUS*) reporter gene, was used to evaluate transgenic effects *via* histochemical GUS staining. Additionally, the recombinant plasmid pCAMBIA2300-*RcLEC1-B*-OE was employed as an alternative reporter system for garlic transformation experiments. *RcLEC1-B* was isolated from PLBs of *Rosa canina*, and its nucleotide sequence has been deposited in GenBank (accession no.: KM115581.1). Notably, previous studies have reported that *RcLEC1-B*-OE alters leaf morphology by impairing cuticle development and reduces plant yield. Vector construction was performed according to methods previously described in the literature (Xu et al., 2019b). Furthermore, the nucleotide sequence of *trichosanthin* (*TCS*) from *Trichosanthes kirilowii* (GenBank accession no.: AF367252.1) was used as a reference for vector construction. The TCS gene was employed as a functional gene to assess the disease resistance of garlic plants transformed with pCAMBIA1300-*TkTCS*-OE and pCAMBIA2300-*TkTCS*-OE.

### 2.7 Establishment of an explant pre-set transformation system mediated by FELBs and RTBs

Explants were submerged in *Agrobacterium* transformation and infiltration medium (TIM), which was prepared with 1/2 MS salts, 1/2 B5 vitamins, 50 g/L sucrose, 0.03% Silwet L-77, and 0.01 mg/L BAP; the medium pH was adjusted to 5.8, and the *Agrobacterium* suspension was adjusted to an optical density at 600 nm (OD_600_) of 0.5-0.6. The suspension containing the immersed explants was gently agitated in darkness for 8–10 minutes. Following infiltration, the explants were carefully blotted dry with sterile absorbent paper and transferred to co-culture medium (C-CM). C-CM was based on MS medium supplemented with 40.0 mg/L acetosyringone (AS), 30.0 g/L sucrose, and 6.5 g/L agar, with a pH of 5.8. Explants were incubated on C-CM in darkness for 48–72 hours.

Subsequently, explants were transferred to FELB induction selective medium (FELBISM) for the induction of translucent mucilaginous callus and FELBs. This medium consisted of MS medium fortified with auxin analogues (either 2,4-D alone, NAA alone, or a combination of 2,4-D and NAA at concentrations of 0, 1.25, 2.5 and 3.75 mg/L), 25 mg/L hygromycin B (Hyg B), 500 mg/L carbenicillin (Carb), and 3.45 g/L gellan gum (pH 5.8). For FELB formation, explants were maintained under strict stress conditions in complete darkness, with no exposure to light allowed.

To generate RTBs, rhizoids developed on explants were transferred to rhizoid induction selective medium (RsISM) and cultured in darkness. This medium was based on MS formulation, supplemented with auxin analogues (2,4-D and/or NAA at their optimal concentrations), 25 mg/L Hyg B, 500 mg/L Carb, and 3.45 g/L gellan gum, with the pH adjusted to 5.8. Subsequently, rhizoid clusters attached to their original explants were transplanted *in situ* onto RTB induction selective medium (RTBISM) and cultured under high light intensity (180 μmol·m^-2^·s^-1^). RTBISM consisted of MS salts, MS vitamins, 25 mg/L Hyg B, 500 mg/L Carb, 3.45 g/L gellan gum (pH 5.8), and thidiazuron (TDZ) at the optimal concentration. Finally, the regenerated plantlets were transferred to RM. For transgenic line screening, 25 mg/L Hyg B was used as the selective agent for plants transformed with the pCAMBIA1300 vector, while DNA-PCR detection was performed to validate transgenic lines obtained with the pBI121 and pCAMBIA2300 vectors.

### 2.8 GUS histochemical assay on longitudinal sections of garlic sprouts of pBI121 garlic plants

A GUS histochemical assay was performed on the longitudinal sections of garlic cloves from pBI121-transformed plants, which were regenerated *via* the FELB and RTB pathways, respectively. The assay was conducted following the method described by Jefferson et al. (1987) with minor modifications. First, the longitudinal sections of garlic cloves were immersed in a staining solution. This staining solution was prepared by dissolving 0.96 mM 5-bromo-4-chloro-3-indolyl β-D-glucuronide (X-Gluc) in dimethylformamide (DMF), which was then added to a 0.1 M PBS solution (pH 7.0). The PBS solution was supplemented with 0.5 mM ethylenediaminetetraacetic acid (EDTA), 5 mM potassium ferricyanide, 5 mM potassium ferrocyanide, 2% methanol, and 0.1% (v/v) Triton X-100. The sections were incubated in this staining solution overnight at 37°C in the dark. After X-Gluc staining, the samples were subjected to de-staining, transparency, and rehydration processes in sequence, as follows: the samples were first rehydrated in a gradient of ethanol solutions (75%, 37.5%, and 18.75%), with each step lasting 30 minutes. Subsequently, they were placed in a solution containing 5% ethanol and 25% glycerol for 30 minutes. Finally, the samples were stored in 50% glycerol for subsequent observation and photography.

### 2.9 DNA extraction and PCR assay of transgenic garlic plants

Genomic DNA was extracted from transgenic garlic plants using the RaPure Plant DNA Mini Kit D3187-03 (Magen Biotech Co., Ltd., Guangzhou, China) according to the manufacturer’s instructions. For PCR amplification of pBI121-transformed plants, the reaction protocol was as follows: initial pre-denaturation at 94 °C for 5 minutes, followed by 35 cycles of denaturation at 94 °C for 45 seconds, annealing at 50 °C for 45 seconds, and extension at 72 °C for 50 seconds. Primers specific to the *GUS* gene were used: forward primer (F): 5’-GCGGGCAACGTCTGGTATC AGCGCG-3’ and reverse primer (R): 5’-GTGCCTTGTCCAGTTGCAACCACCT-3’. The amplified fragment was 500 bp in length. A final extension step was performed at 72 °C for 10 minutes to ensure complete product elongation.

For PCR detection of pCAMBIA2300-*RcLEC1-B*-OE-transformed plants, the protocol was as follows: initial pre-denaturation at 94°C for 5 minutes, followed by 35 cycles consisting of denaturation at 94°C for 30 seconds, annealing at 55°C for 30 seconds, and extension at 72°C for 1 minute. Primers targeting *RcLEC1-B* were: forward primer 5’-ATGAGGCTGGCTGAGATCAC CCACA-3’ and reverse primer 5’-TCACTTATTGTTATCATCATACTGG-3’; the open reading frame (ORF) of *RcLEC1-B* is 642 bp. A final extension step was conducted at 72°C for 10 minutes (Xu et al., 2019a, Xu et al., 2019b).

For pCAMBIA1300-*TkTCS*-OE and pCAMBIA2300-*TkTCS*-OE garlic plants, PCR amplification was performed with the following protocol: pre-denaturation at 94°C for 5 minutes, followed by 35 cycles of denaturation at 94°C for 45 seconds, annealing at 58°C for 45 seconds, and extension at 72°C for 1 minute. Primers specific to *TkTCS* were: forward primer 5’-ATGATCAGA TTCTTAGTCCTCTCTT-3’ and reverse primer 5’-CTAAATAGCATAACTTCCACATCCA-3’; the ORF of *TkTCS* is 870 bp. A final extension step was carried out at 72°C for 10 minutes. All amplified products were separated on 1.0% agarose gels and detected by agarose gel electrophoresis. The PCR products of *GUS* (from pBI121), *RcLEC1-B* and *TkTCS* were sequenced by Sangon Biotech (Zhengzhou, China).

### 2.10 Microscopic analysis of the interaction between *P. chrysogenum*, *P. porri* and *TkTCS*-OE garlic plants

The pathogenicity test was conducted using the fungal spore suspension inoculation method. Specifically, spore suspensions of *Penicillium chrysogenum* (ACCC31569) and *Phytophthora porri* (ACCC30382) were cultured in Luria-Bertani (LB) liquid medium at 28°C with shaking at 180 rpm for 8 hours. The spores were then harvested *via* centrifugation at 5000×g for 10 minutes. The spore pellets were resuspended in a 0.05% Tween-20 aqueous solution to adjust the concentration to 10^4–^10^6^ colony forming units (CFU) per milliliter, which was used for subsequent infection assays.

The endogenous peroxidase-dependent 3,3-diaminobenzidine (DAB) assay was employed to detect the production of H_2_O_2_ in plants, as described by Thordal-Christensen et al. (2010). To investigate H_2_O_2_ accumulation, garlic cloves were harvested at 17 days post-inoculation with *P. chrysogenum* and 28 days post-inoculation with *P. porri*. These garlic cloves were immersed in a DAB solution (1 mg/ml, pH 3.8) for 8–12 hours until visible DAB staining appeared on the surface. Subsequently, the DAB-stained garlic cloves were fixed and stained with 0.6% Coomassie Brilliant Blue R250 in methanol (w/v), following the protocol adapted from Li et al (2006). The epidermal layers of the garlic cloves were observed under a differential interference contrast microscope (BX61, Olympus Corporation, Japan), and images were captured using a color video camera integrated with image analysis software (Image-Pro Plus 4.1, Media Cybernetics, L.P.). For these examinations, five biological replicates of microscopic samples were used for both *TkTCS*-OE garlic plants and wild-type plants.

### 2.11 Phenotypic trait analysis

Images of the translucent mucilaginous callus and FELBs at different developmental stage, as well as images of rhizoids and RTBs at different developmental stages and GUS histochemical staining results, were captured using a digital camera (EOS 600D, Canon Inc., Japan) coupled with a stereomicroscope (SMZ25, Nikon Corporation, Japan). Plant phenotypic features, including color, leaf curvature, and tillering capacity, were meticulously observed, investigated, and recorded using the EOS 600D camera.

### 2.12 Statistical analysis

All experiments were conducted with three independent biological replicates. To evaluate the induction frequencies of translucent mucilaginous callus, FELBs, rhizoids, and RTBs, 100 Petri dishes were prepared for each biological replicate, with 10 explants inoculated per dish, resulting in a total of 1000 explants per replicate. These 100 petri dishes were further randomly divided into 10 treatment subgroups. For each subgroup (consisting of 10 petri dishes), the 100 explants were defined as one basic statistical unit, and the mean value was calculated accordingly. Across the three independent biological replicates, there were 30 treatment subgroups in total, with a final sample size of n = 30. All three replicates were integrated for unified statistical analysis.

All data were analyzed using the generalized linear model (GLM). When significant interactive effects were observed among PGRs type (2,4-D alone, NAA alone, or a combination of 2,4-D and NAA), PGRs concentration, and explant type, *post hoc* pairwise comparisons were further conducted as follows: 1) Comparison among different concentrations under the same PGRs type; 2) Comparison among different PGRs types at the same concentration level; 3) Comparison among different explant types at the same concentration level. All statistical analyses were performed using IBM SPSS Statistics 19.0 (SPSS Inc., Chicago, IL, USA).

## 3 Results

### 3.1 Combined application of 2,4-D and NAA triggered formation of translucent mucilaginous callus and FELBs

Among all tested explants, including root segments, garlic cloves, garlic clove discs, stem tips, stem segments, leaf segments, and garlic scape segments, only three types were capable of inducing translucent mucilaginous substances: root segments, stem tips, and garlic scape segments. No callus was formed when 2,4-D and NAA were omitted from the induction medium, indicating that the combination of PGRs was indispensable for callus initiation in garlic. Wald χ² model analysis was performed to identify factors modulating transparent mucilaginous callus induction from garlic root segments, stem tip, and scape segment explants. Highly significant main effects of PGR type (df = 2, Wald χ² = 20538.041, *P* < 0.001; df = 2, Wald χ² = 75.219, *P* < 0.001; df = 2, Wald χ² = 1961.779, *P* < 0.001) and PGR concentration (df = 3, Wald χ² = 7854.678, *P* < 0.001; df = 3, Wald χ² = 186.271, *P* < 0.001; df = 3, Wald χ² = 1570.579, *P* < 0.001) were detected for callus induction rates. A prominent, statistically significant interaction between PGR type and concentration was also observed (df = 6, Wald χ² = 12606.172, *P* < 0.001; df = 6, Wald χ² = 49.170, P < 0.001; df = 6, Wald χ² = 2095.269, *P* < 0.001), demonstrating that the callogenic response to elevated PGR concentrations differed substantially among three PGR regimes (2,4-D single application, NAA single application, and combined 2,4-D + NAA). Four PGR concentration gradients (0, 1.25, 2.5, 3.75 mg L⁻¹) were implemented in the trial (**Tables 1**–**3**). Single supplementation with either 2,4-D or NAA at graded concentrations (1.25, 2.5, and 3.75 mg/L) only triggered limited callus formation, suggesting that individual application of 2,4-D or NAA was insufficient to support efficient somatic embryogenesis. In contrast, the combined use of 2,4-D and NAA, both at 2.5 mg/L, produced a markedly higher induction rate of translucent mucilaginous callus in root segments, reaching 98.433%. This value was significantly greater than that obtained with other concentration combinations or alternative explant types (**Tables 4**–**6**). Furthermore, comparative analysis of FELB induction frequency among root segments, stem tips and garlic scape segments showed that only root segments were competent for FELB formation (**Table 7**). Wald χ² testing was applied to quantify factors controlling FELB induction rates from garlic explants incubated under dark culture. Both PGR concentration (df = 3, Wald χ² = 14623.003, *P* < 0.001) and explant type (df = 2, Wald χ² = 45981.511, *P* < 0.001) imposed highly significant main effects on FELB formation efficiency. A strong, statistically significant interaction between PGR concentration and explant type was also detected (df = 6, Wald χ² = 29246.006, *P* < 0.001), showing that the promotive effect of 2,4-D + NAA dosage on FELB induction varied substantially across three tested explant materials (garlic root segments, stem tips, and scape segments). Four combined 2,4-D and NAA concentrations (0, 1.25, 2.5, 3.75 mg L⁻¹) were included in the single-factor gradient setup (**Table 8**). Under the combined treatment of 2,4-D and NAA both at 2.5 mg/L, root segments produced a markedly higher FELB induction frequency of 96.333%, which was significantly superior to those obtained at lower concentrations (46.633% and 31.467%, respectively; **Table 7**).

**Table 1:** Model effect test of PGRs types and concentrations on induction rate of transparent mucilaginous callus from garlic root segments under conditions.

Variables	df	Wald χ²	P value
PGRs types	2	20538.041	< 0.001
PGRs concentrations	3	7854.678	< 0.001
PGRs types × PGRs concentrations	6	12606.172	< 0.001

PGRs types included 2,4-D alone, NAA alone, and a combination of 2,4-D and NAA. PGRs concentrations were 0, 1.25, 2.5, and 3.75 mg/L.

**Table 2:** Model effect test of PGRs types and concentrations on induction rate of transparent mucilaginous callus from garlic stem tips under conditions.

Variables	df	Wald χ²	P value
PGRs types	2	75.219	< 0.001
PGRs concentrations	3	186.271	< 0.001
PGRs types × PGRs concentrations	6	49.170	< 0.001

PGRs types included 2,4-D alone, NAA alone, and a combination of 2,4-D and NAA. PGRs concentrations were 0, 1.25, 2.5, and 3.75 mg/L.

**Table 3:** Model effect test of PGRs types and concentrations on induction rate of transparent mucilaginous callus from garlic scape segments under conditions.

Variables	df	Wald χ²	P value
PGRs types	2	1961.779	< 0.001
PGRs concentrations	3	1570.579	< 0.001
PGRs types × PGRs concentrations	6	2095.269	< 0.001

PGRs types included 2,4-D alone, NAA alone, and a combination of 2,4-D and NAA. PGRs concentrations were 0, 1.25, 2.5, and 3.75 mg/L.

**Table 4:** Interactive effects of PGRs types and concentrations on induction rate of transparent mucilaginous callus from garlic root segments under conditions.

2,4-D (mg/L)	NAA (mg/L)	Induction rate (%) of translucent mucilaginous callus
0		0.000 ± 0.000 Ca
1.25		1.600 ± 0.252 Bb
2.50		3.000 ± 0.339 Ab
3.75		1.167 ± 0.235 Bb
	0	0.000 ± 0.000 Ba
	1.25	3.267 ± 0.547 Ab
	2.50	4.200 ± 0.784 Ab
	3.75	1.033 ± 0.217 Bb
0	0	0.000 ± 0.000 Da
1.25	1.25	49.067 ± 0.980 Ba
2.50	2.50	98.433 ± 0.546 Aa
3.75	3.75	33.800 ± 0.777 Ca

PGRs types included 2,4-D alone, NAA alone, and a combination of 2,4-D and NAA. PGRs concentrations were 0, 1.25, 2.5, and 3.75 mg/L. The induction rate of translucent mucilaginous callus was calculated as the ratio of the total number of translucent mucilaginous callus induced in each treatment to the total number of inoculated garlic root segment explants (mean ± SE, n = 30). Uppercase letters indicate significant differences among different hormone concentrations within each hormone combination type, while lowercase letters indicate significant differences among different hormone combination types at each hormone concentration.

**Table 5:** Interactive effects of PGRs types and concentrations on induction rate of transparent mucilaginous callus from garlic stem tips under conditions.

2,4-D (mg/L)	NAA (mg/L)	Induction rate (%) of translucent mucilaginous callus
0		0.000 ± 0.000 Ba
1.25		0.867 ± 0.190 Ac
2.50		1.500 ± 0.157 Ab
3.75		0.767 ± 0.196 Ab
	0	0.000 ± 0.000 Ca
	1.25	2.033 ± 0.334 Bb
	2.50	5.733 ± 0.867 Aa
	3.75	2.667 ± 0.424 Ba
0	0	0.000 ± 0.000 Da
1.25	1.25	3.900 ± 0.485 Ba
2.50	2.50	5.767 ± 0.621 Aa
3.75	3.75	2.467 ± 0.351 Ca

PGRs types included 2,4-D alone, NAA alone, and a combination of 2,4-D and NAA. PGRs concentrations were 0, 1.25, 2.5, and 3.75 mg/L. The induction rate of translucent mucilaginous callus was calculated as the ratio of the total number of translucent mucilaginous callus induced in each treatment to the total number of inoculated garlic stem tips explants (mean ± SE, n = 30). Uppercase letters indicate significant differences among different hormone concentrations within each hormone combination type, while lowercase letters indicate significant differences among different hormone combination types at each hormone concentration.

**Table 6:** Interactive effects of PGRs types and concentrations on induction rate of transparent mucilaginous callus from garlic scape segments under conditions.

2,4-D (mg/L)	NAA (mg/L)	Induction rate (%) of translucent mucilaginous callus
0		0.000 ± 0.000 Ba
1.25		1.067 ± 0.257 Ab
2.50		1.833 ± 0.259 Ac
3.75		1.333 ± 0.260 Ab
	0	0.000 ± 0.000 Ca
	1.25	2.100 ± 0.323 Ab
	2.50	3.133 ± 0.361 Ab
	3.75	1.167 ± 0.292 Bb
0	0	0.000 ± 0.000 Da
1.25	1.25	5.933 ± 0.346 Ca
2.50	2.50	30.000 ± 0.974 Aa
3.75	3.75	10.000 ± 0.392 Ba

PGRs types included 2,4-D alone, NAA alone, and a combination of 2,4-D and NAA. PGRs concentrations were 0, 1.25, 2.5, and 3.75 mg/L. The induction rate of translucent mucilaginous callus was calculated as the ratio of the total number of translucent mucilaginous callus induced in each treatment to the total number of inoculated garlic scape segments explants (mean ± SE, n = 30). Uppercase letters indicate significant differences among different hormone concentrations within each hormone combination type, while lowercase letters indicate significant differences among different hormone combination types at each hormone concentration.

**Table 7:** Interactive effects of PGRs concentrations and explant types on FELBs induction rate under dark conditions.

2,4-D (mg/L)	NAA (mg/L)	Frequency (%) of FELB induction		
		Root segments	Stem tips	Garlic scape segments
0	0	0.000 ± 0.000 Da	0.000 ± 0.000 Aa	0.000 ± 0.000 Aa
1.25	1.25	46.633 ± 0.753 Ba	0.000 ± 0.000 Ab	0.000 ± 0.000 Ab
2.50	2.50	96.333 ± 0.787 Aa	0.000 ± 0.000 Ab	0.000 ± 0.000 Ab
3.75	3.75	31.467 ± 0.428 Ca	0.000 ± 0.000 Ab	0.000 ± 0.000 Ab

PGRs concentrations (combination of 2,4-D and NAA) were 0, 1.25, 2.5, and 3.75 mg/L. Explant types were garlic root segments, stem tips and scape segments. The FELBs induction rate was calculated as the ratio of the total number of FELBs induced in each treatment to the total number of inoculated garlic explants (mean ± SE, n = 30). Uppercase letters indicate significant differences among different hormone concentrations within each hormone combination type, while lowercase letters indicate significant differences among different hormone combination types at each hormone concentration.

**Table 8:** Model effect test of PGRs concentrations and explant types on FELBs induction rate under dark conditions.

Variables	df	Wald χ²	P value
PGRs concentrations	3	14623.003	< 0.001
Explant types	2	45981.511	< 0.001
PGRs concentrations × Explants types	6	29246.006	< 0.001

PGRs concentrations (combination of 2,4-D and NAA) were 0, 1.25, 2.5, and 3.75 mg/L. Explant types were garlic root segments, stem tips and scape segments.

**Figure 2:**
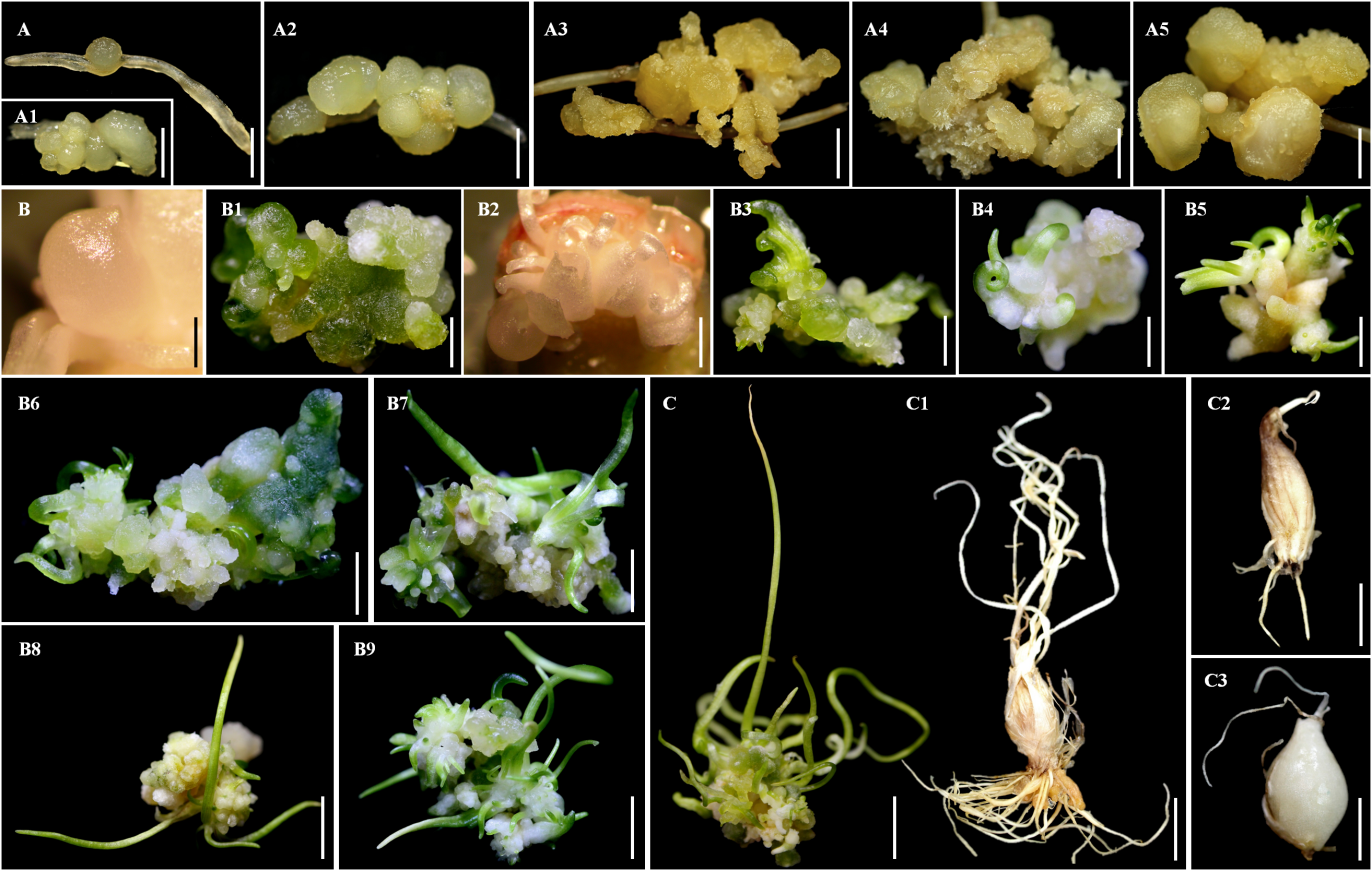
Development of FELB from root segments in garlic. **(A-A5)** Ontogenetic progression of translucent mucilaginous callus and FELB induction from root explants. **(A)** Translucent mucilaginous callus with a single globular-shaped embryo induced from root explant. **(A1-A2)** Early-stage clustered FELB structures. **(A3-A5)** Mature FELBs with irregular morphology and desiccated surfaces characteristics. **(B-B9)** Germination process of FELBs under photoperiod incubation conditions. **(B)** Isolated intact FELB. **(B1)** Color transition of FELBs from pale yellow to bright green. **(B2-B3)** Initial germination stage of FELB. **(B4-B6)** Middle germination stage of FELB. **(B7-B9, C)** Late germination stage of FELB. **(C1-C3)** Formation and growth of single-head garlic bulbs derived from FELB-regenerated plantlets. Scale bars **(A-A5, B-B9, C-C1)** = 1 cm, scale bars **(C2, C3)** = 0.5 cm.

Under dark culture conditions, obvious mucilaginous callus formed on the surface of root segments after 15 days post inoculation (dpi) under combined 2,4-D and NAA treatment. After 25 dpi, individual globular early FELB embryos emerged from the callus (**Figure 2A**). Cluster-like FELB structures subsequently formed at 45 dpi (**Figure 2A1, A2**). During FELB induction, both the number and size of embryos increased progressively, accompanied by irregular morphological changes and gradual desiccation of the surface (**Figure 2A3, A4**). The abundance of FELBs reached its peak after 90 dpi (**Figure 2A5**). FELB embryos were capable of subculture to generate secondary FELBs, whereas early FELB embryos without subculture gradually underwent senescence. Consistent with previous reports on FELB structures in dicotyledonous species including *Solanum nigrum* (Xu et al., 2014) and *Rorippa indica* (Xu et al., 2016), garlic FELB embryos failed to germinate spontaneously. When cultured under a light intensity of 180 µmol·m^-2^s^-1^, FELB embryos gradually turned from pale yellow to bright green (**Figure 2B1**), and mature FELBs were further induced to undergo plant regeneration (**Figure 2B4–B6**). PGR regulation was essential for FELB germination in garlic. BAP exhibited a strong inductive effect on FELB embryo germination, with 5 mg/L BAP identified as the optimal concentration, achieving a germination rate of 100% (**Figure 2B7–B9**). Furthermore, FELB-regenerated plantlets could develop normally into single-head garlic plantlets under *in vitro* culture conditions (**Figure 2C1–C3**).

**Figure 3:**
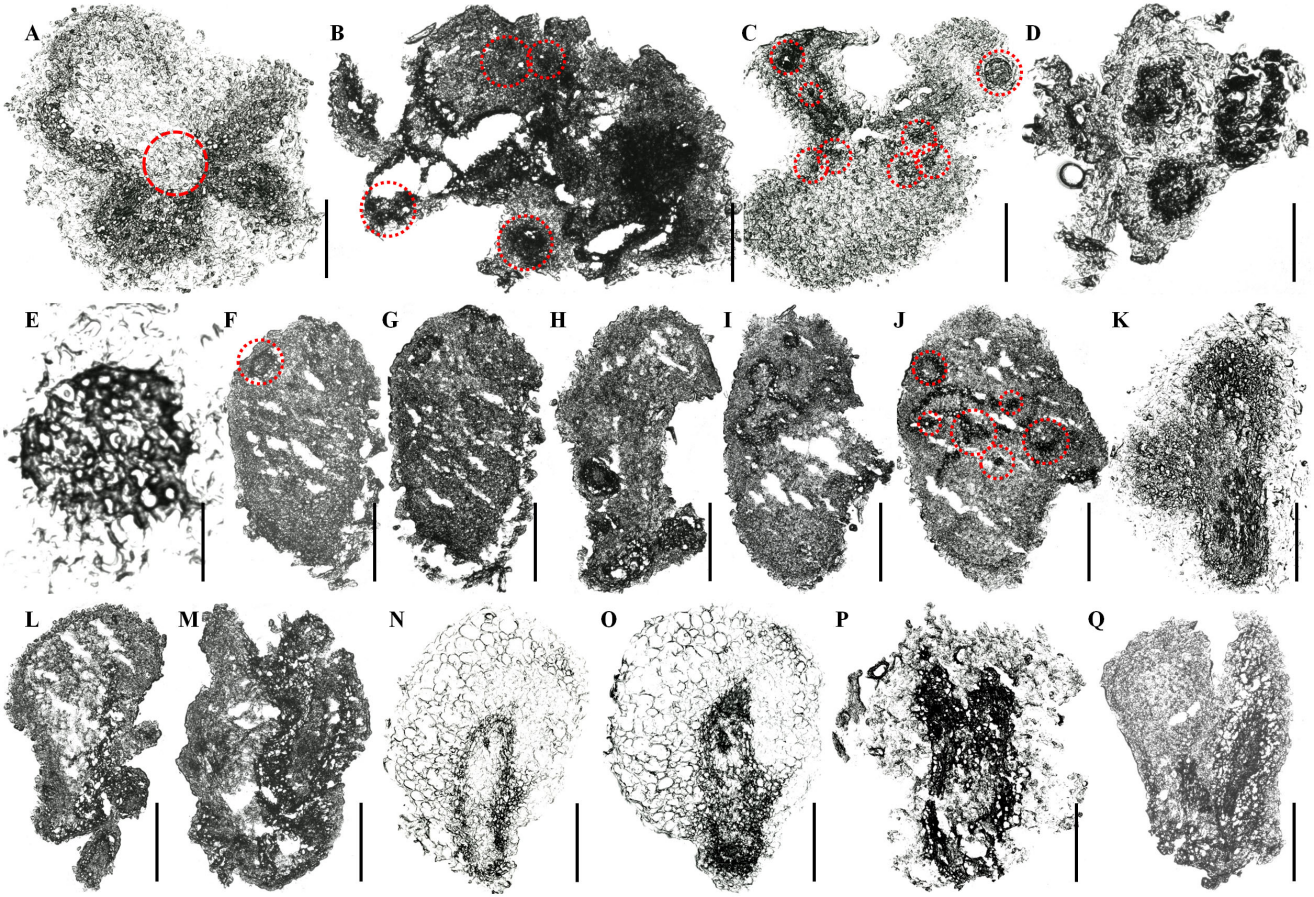
Microstructural observation of FELB at different developmental stages in garlic via frozen sectioning. **(A)** Translucent mucilaginous callus induced from root segment explant, showing cortical tissues. **(B)** Potential FCDZs within callus containing proembryos. **(C, D)** FELBs bearing multiple embryoids at distinct developmental stages, with abundant early globular-stage embryos. **(E)** Isolated single globular-shaped embryo. **(F-M)** Embryos at torpedo transitional stage. **(N-Q)** Mature FELB-derived embryos. Scale bars **(A, C, D, F-Q)** = 100 µm, scale bar **(B)** = 50 µm, scale bar **(E)** = 25 µm.

Phytocytohistological analyses of the FELB development process revealed that FELB pro-embryos were formed in the cortical region of garlic root segments (**Figure 3A**), which subsequently developed into a fast cell division zones (FCDZs). The FELB embryo development mainly proceeded through two distinct stages: the globular embryo stage (**Figures 3C–E**) and the torpedo-shaped transition embryo stage (**Figures 3F–M**). At the mature stage, the vascular tissue development in FELB-induced embryoids was highly similar to that of dicotyledonous plants (**Figures 3N–Q**) (Xu et al., 2014, Xu et al., 2016).

### 3.2 Rhizoids were efficiently induced from root segments under NAA and sucrose semistarvation in darkness

*In vitro* culture experiments demonstrated that among various garlic explants, including garlic cloves, garlic clove discs, stem tips, stem segments, leaf segments, and garlic scape segments, only root segments exhibited morphogenetic capacity for rhizoid formation. NAA was essential for garlic rhizoid induction, as no rhizoids were formed in media without NAA supplementation. Wald χ² generalized linear model testing was performed to evaluate factors regulating rhizoid formation from garlic root explants cultured in darkness with half-strength sucrose. Both PGR type (df = 1, Wald χ² = 4704.676, *P* < 0.001) and PGR concentration (df = 3, Wald χ² = 5643.409, *P* < 0.001) exerted highly significant main effects on rhizoid induction numbers. A strong, statistically significant interaction between PGR type and concentration was also detected (df = 3, Wald χ² = 2431.184, *P* < 0.001), demonstrating that the rhizogenic response to increasing PGR dosage varied between treatments supplied with sole NAA and a 2,4-D + NAA combination. Treatments comprised two PGR formulations and four concentration levels (0, 5.0, 10.0, 15.0 mg L⁻¹) (**Table 9**). Among the tested NAA concentrations, 10.0 mg/L yielded the highest average number of rhizoids per explant (63.5), which was thus identified as the optimal concentration. Notably, the synergistic effect of 2,4-D and NAA on rhizoid induction was significantly weaker than that of NAA alone, with the maximum average number of rhizoids per explant reaching only 13.533-approximately one-fifth of the count induced by NAA alone (**Table 10**). Generalized linear model Wald χ² testing was conducted to assess drivers of rhizoid production from garlic root segment explants incubated in darkness with full-strength sucrose. Highly significant main effects of PGR type (df = 1, Wald χ² = 1322.750, *P* < 0.001) and PGR concentration (df = 3, Wald χ² = 2067.182, *P* < 0.001) were detected for rhizoid induction numbers. A significant interactive effect between PGR type and concentration was also identified (df = 3, Wald χ² = 527.314, *P* < 0.001), indicating that the rhizogenic response to rising PGR concentrations varied between treatments with single NAA and combined 2,4-D + NAA. The experiment incorporated two PGR formulations and four concentration levels (0, 5.0, 10.0, 15.0 mg L⁻¹) (**Table 11**). Additionally, an interesting phenomenon was observed: sucrose semistarvation (half the normal concentration) increased the number of rhizoids induced per root segment compared to the standard sucrose concentration (**Table 12**). Collectively, these results indicated that the optimal rhizoid induction combination was 10.0 mg/L NAA supplemented with half-strength sucrose (**Table 10**).

**Table 9:** Model effect test of PGRs types and concentrations on rhizoid induction numbers from garlic root segments explants under dark conditions with half-strength sucrose supplementation.

Variables	df	Wald χ²	P value
PGRs types	1	4704.676	< 0.001
PGRs concentrations	3	5643.409	< 0.001
PGRs types × PGRs concentrations	3	2431.184	< 0.001

PGRs types included NAA alone and a combination of 2,4-D and NAA. PGRs concentrations were 0, 5.0, 10.0 and 15.0 mg/L.

**Table 10:** Interactive effects of PGRs types and concentrations on rhizoid induction numbers from garlic root segments explants under dark conditions with half-strength sucrose supplementation.

NAA (mg/L)	2,4-D (mg/L)	Average induction numbers of rhizoid
0		0.000 ± 0.000 Da
5.0		40.700 ± 0.788 Ba
10.0		63.500 ± 0.604 Aa
15.0		21.933 ± 0.772 Ca
0	0	0.000 ± 0.000 Da
5.0	5.0	5.967 ± 0.461 Bb
10.0	10.0	13.533 ± 0.571 Ab
15.0	15.0	3.267 ± 0.479 Cb

PGRs types included NAA alone and a combination of 2,4-D and NAA. PGRs concentrations were 0, 5.0, 10.0 and 15.0 mg/L. The average induction numbers of rhizoids was calculated as the ratio of the total number of rhizoids induced in each treatment to the total number of inoculated garlic root segments explants under dark conditions with half-strength sucrose supplementation (mean ± SE, n = 30). Uppercase letters indicate significant differences among different hormone concentrations within each hormone combination type, while lowercase letters indicate significant differences among different hormone combination types at each hormone concentration.

**Table 11:** Model effect test of PGRs types and concentrations on rhizoid induction numbers from garlic root segments explants under dark conditions with full-strength sucrose supplementation.

Variables	df	Wald χ²	P value
PGRs types	1	1322.750	< 0.001
PGRs concentrations	3	2067.182	< 0.001
PGRs types × PGRs concentrations	3	527.314	< 0.001

PGRs types included NAA alone and a combination of 2,4-D and NAA. PGRs concentrations were 0, 5.0, 10.0 and 15.0 mg/L.

**Table 12:** Interactive effects of PGRs types and concentrations on rhizoid induction numbers from garlic root segments explants under dark conditions with full-strength sucrose supplementation.

NAA (mg/L)	2,4-D (mg/L)	Average induction numbers of rhizoid
0		0.000 ± 0.000 Da
5.0		19.800 ± 0.845 Ba
10.0		31.467 ± 0.698 Aa
15.0		13.700 ± 0.421 Ca
0	0	0.000 ± 0.000 Ca
5.0	5.0	3.200 ± 0.427 Bb
10.0	10.0	10.967 ± 0.471 Ab
15.0	15.0	2.000 ± 0.284 Bb

PGRs types included NAA alone and a combination of 2,4-D and NAA. PGRs concentrations were 0, 5.0, 10.0 and 15.0 mg/L. The average induction numbers of rhizoids was calculated as the ratio of the total number of rhizoids induced in each treatment to the total number of inoculated garlic root segments explants under dark conditions with full-strength sucrose supplementation (mean ± SE, n = 30). Uppercase letters indicate significant differences among different hormone concentrations within each hormone combination type, while lowercase letters indicate significant differences among different hormone combination types at each hormone concentration.

**Figure 4:**
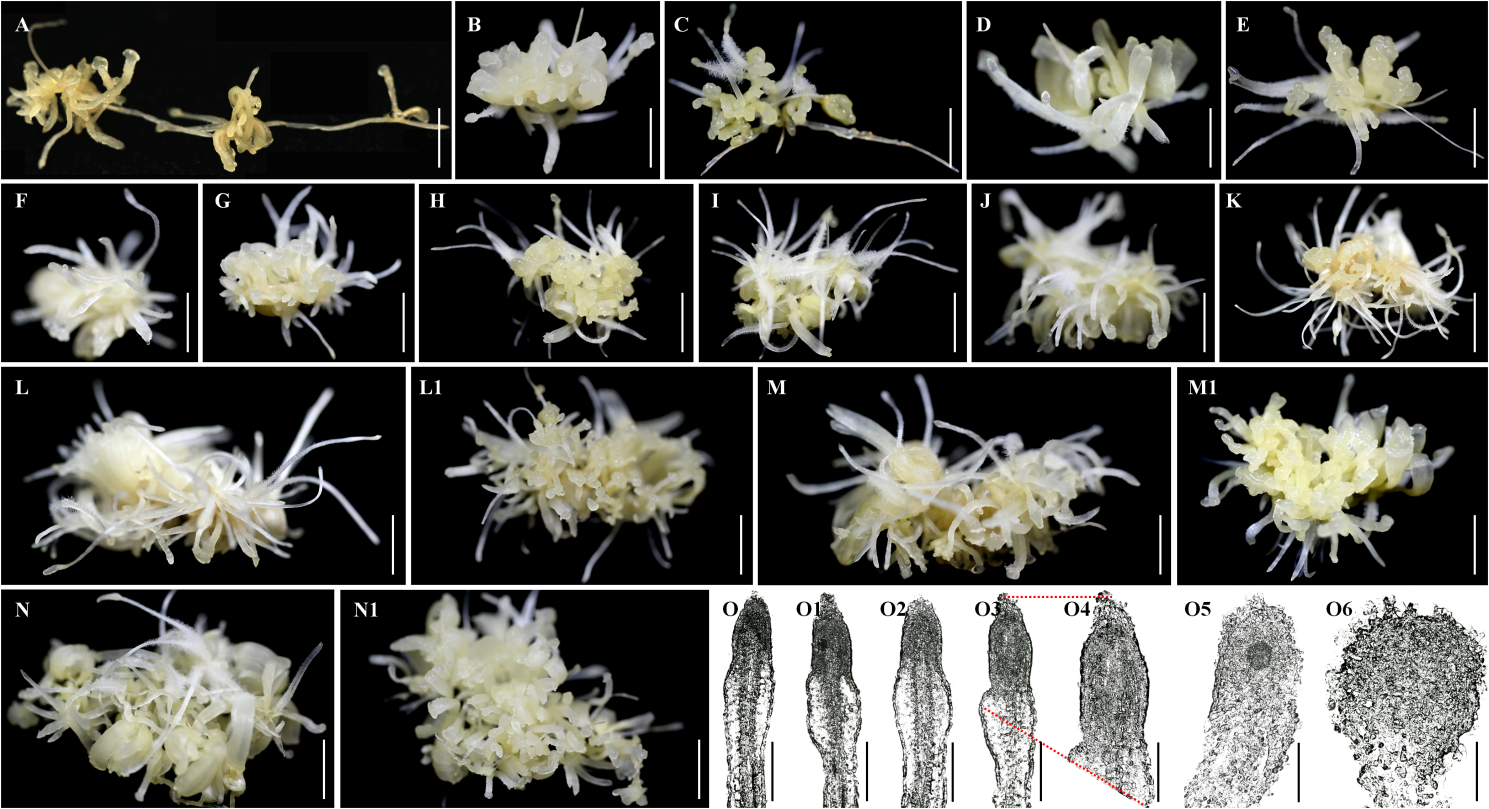
Induction and developmental progression of rhizoids from garlic root segments. **(A-E)** Early-stage rhizoids at 10, 15, 20, 25, and 30 dpi, corresponding to panels **(A-E)**, respectively. **(F-K)** Middle-stage rhizoids at 35, 40, 45, 50, 55, and 60 dpi, corresponding to panels **(F-K)**, respectively. **(L-N1)** Mature-stage rhizoids, panels **(L, L1, M, M1, N, N1)** show the frontal and lateral views of rhizoids at 70, 80, and 90 dpi, respectively. **(O-O2)** Longitudinal sections of early-stage rhizoids at 10, 20, and 30 dpi [**(O, O1, O2)**, respectively). **(O3-O4)** Longitudinal sections of rhizoids at 60 dpi; **(O4)** is a magnified view of **(O3)**, showing the rhizoid cap structure. **(O5)** Longitudinal section of rhizoids at 80 dpi, displaying the cortex and FCDZs. **(O6)** Longitudinal frozen section of rhizoids at 90 dpi, showing the cortical tissue and inflated rhizoid cap. Scale bars (A-N, L1, M1, N1) = 1 cm, scale bars **(O-O3)** = 2 mm, scale bars **(O4-O6)** = 1 mm.

In the early stage of induction, a few white hair-like rhizoids began to radiate from specific sites on root segment explants at 10 dpi (**Figure 4A**). As induction progressed, the original structural integrity of root segments gradually diminished (**Figure 4F–N1**). Rhizoids derived from root segments exhibited non-directional growth and could be roughly classified into two morphological types: one type protruded above the induction medium, appearing relatively long, thin, and fragile (**Figures 4L, M**); the other extended into the medium, being shorter but sturdier (**Figure 4L1, M1**). Rhizoids matured after 90 dpi and were then competent for RTB induction. In the absence of RTB induction, rhizoids gradually senesced, eventually turning brown and dying, with a maximum survival period of approximately 150 days. Longitudinal frozen section analysis of rhizoid development showed that by 60 dpi, the rhizoid cap region, together with the underlying meristem zone-like and elongation zone-like areas, had expanded significantly (**Figure 4O–O4**).

The 80 dpi time point marked a critical turning point, during which the entire apical structure of rhizoids expanded synchronously (**Figure 4O5**). Notably, FCDZs-the signature structures responsible for the spatial and physical isolation of RTBs-were found to emerge earlier at the rhizoid stage rather than the RTB stage in this study (**Figure 4O5**). By 90 dpi, the rhizoid apex had become significantly enlarged (**Figure 4N, N1, O6**).

### 3.3 Embryoid of RTBs derived from cortex cells and germination under the combined induction of TDZ and high light

**Figure 5:**
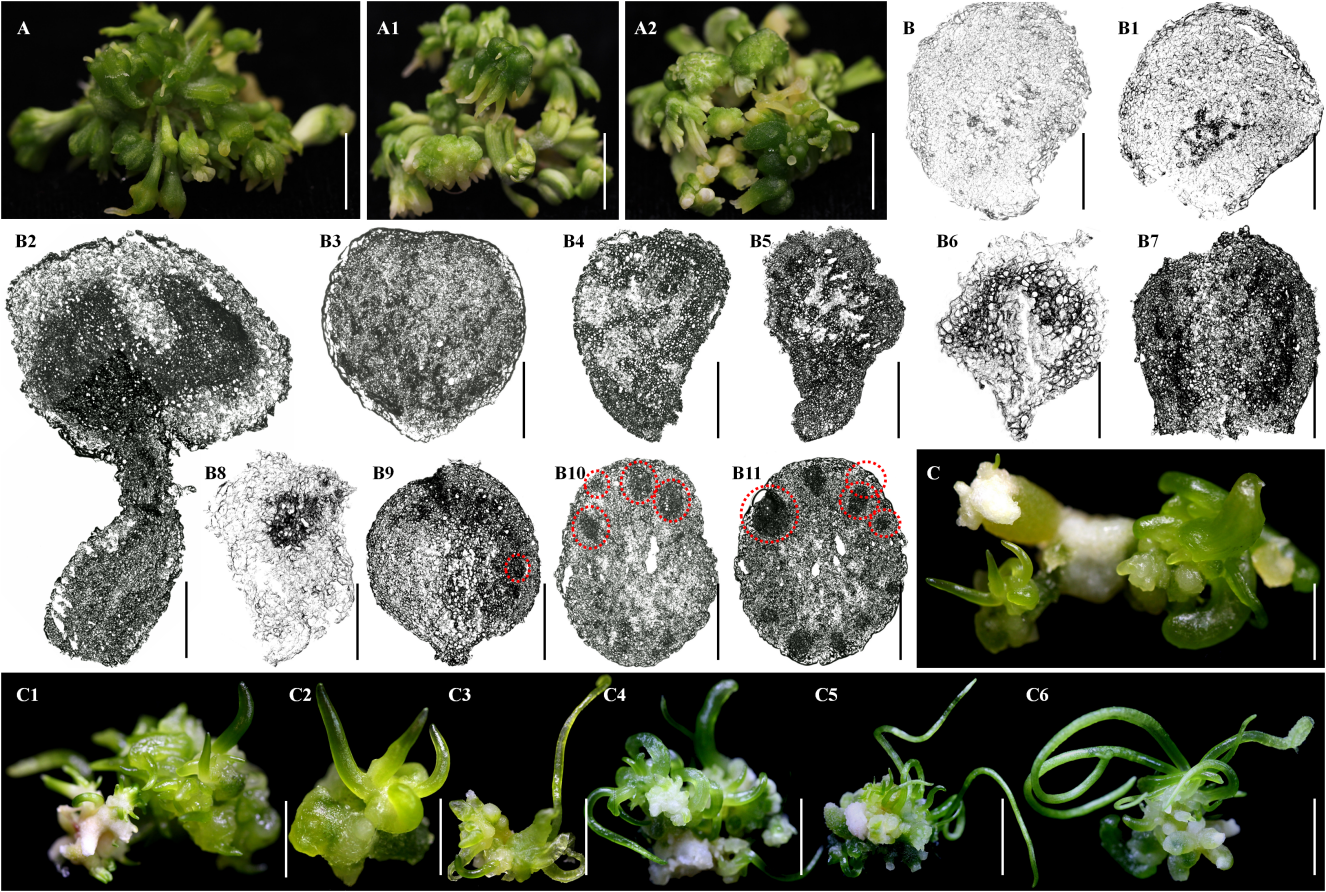
Morphological characterization and developmental observation of RTBs induced from rhizoids of garlic root segments. (**(A)**-A2) RTB structure induced from root segment explants. **(A)** Early-stage RTB at 20 dpt. **(A1)** Middle-stage RTB at 35 dpt. **(A2)** Mature-stage RTB at 50 dpt. (**(B)**-**B11**) Longitudinal frozen sections of RTBs at consecutive developmental stages. **(B-B1)** RTB showing cortical tissue and potential cell division pool at 15 dpt. **(B2)** RTB with rudimentary rhizoids primordia at 20 dpt. **(B3-B4)** RTBs containing abundant pro-embryos at 25 dpt. **(B5-B6)** Swollen RTBs with irregular morphology at 30 dpt. **(B7-B8)** RTBs at the early globular embryo developmental stage at 35 dpt. **(B9-B11)** RTBs bearing torpedo-stage transition embryos at 50 dpt. **(C-C6)** Regeneration process of multiple plantlets derived from *in vitro*-developed RTBs. Scale bars **(A-A2)** = 2 mm, scale bars **(B-B11)** = 300 μm, scale bars **(C-C3)** = 1 cm; scale bars **(C4-C6)** = 2 cm.

Wald χ² analysis revealed that TDZ concentration significantly affected RTB induction numbers of garlic rhizoids under light (df = 3, Wald χ² = 5479.764, *P* < 0.001). Four TDZ levels (0, 10.0, 20.0, 30.0 mg/L) were included in the single-factor experimental design (**Table 13**). Optimization of TDZ supplementation showed that 20.0 mg/L TDZ induced a significantly higher number of RTBs compared with 10.0 and 30.0 mg/L TDZ (**Table 14**). Specifically, 44.067 rhizoids in the 20.0 mg/L TDZ group developed into green, irregular RTBs (**Figure 5A–A2**), which was slightly higher than the average numbers of 24.433 and 14.167 observed in the 10.0 and 30.0 mg/L TDZ groups, respectively. Notably, no RTBs were induced in the medium without TDZ supplementation (**Table 14**).

**Table 13:** Model effect test of TDZ concentrations on RTBs induction numbers from garlic rhizoids under light conditions.

Variables	df	Wald χ²	P value
PGRs concentration	3	5479.764	< 0.001

TDZ concentrations were 0, 10.0, 20.0 and 30.0 mg/L.

**Table 14:** Interactive effects of TDZ concentrations on RTBs induction numbers from garlic rhizoids under light conditions.

TDZ (mg/L)	Average induction numbers of RTBs
0	0.000 ± 0.000 D
10.0	24.433 ± 0.471 B
20.0	44.067 ± 0.518 A
30.0	14.167 ± 0.536 C

TDZ concentrations were 0, 10.0, 20.0 and 30.0 mg/L. The average induction numbers of RTBs was calculated as the ratio of the total number of rhizoids induced in each treatment to the total number of inoculated garlic explant under light conditions (mean ± SE, n = 30). Uppercase letters indicate significant differences among different hormone concentrations within each hormone combination type.

RTBs generally underwent three distinct developmental stages: the early stage (approximately 20 days post transfer, dpt) (**Figure 5A**), the middle stage (roughly 35 dpt) (**Figure 5A1**), and the mature stage (around 50 dpt) (**Figure 5A2**). Longitudinal frozen sections of RTBs at different developmental stages revealed that potential cell division pools (**Figure 5B–B2**), abundant pro-embryos (**Figure 5B3, B4**), early globular-shaped embryos (**Figure 5B7, B8**), and torpedo-shaped transition embryos (**Figure 5B9–B11**) first emerged in cortical cells at 50 dpt. A single RTB could contain multiple embryoids at various developmental stages (**Figure 5B10, B11**). Once mature, these embryoids broke through the epidermis of the RTBs and developed into multiple new plants (**Figure 5C**). When *in vitro* RTBs were cultured on MS medium supplemented with 2.5 mg/L BAP under a light intensity of 180 µmol·m^-^²s^-^¹, multiple regenerated plantlets were formed, with a regeneration rate exceeding 95% (**Figure 5C4–C6**).

### 3.4 Construction of a transgenic system using the EPST method

**Figure 6:**
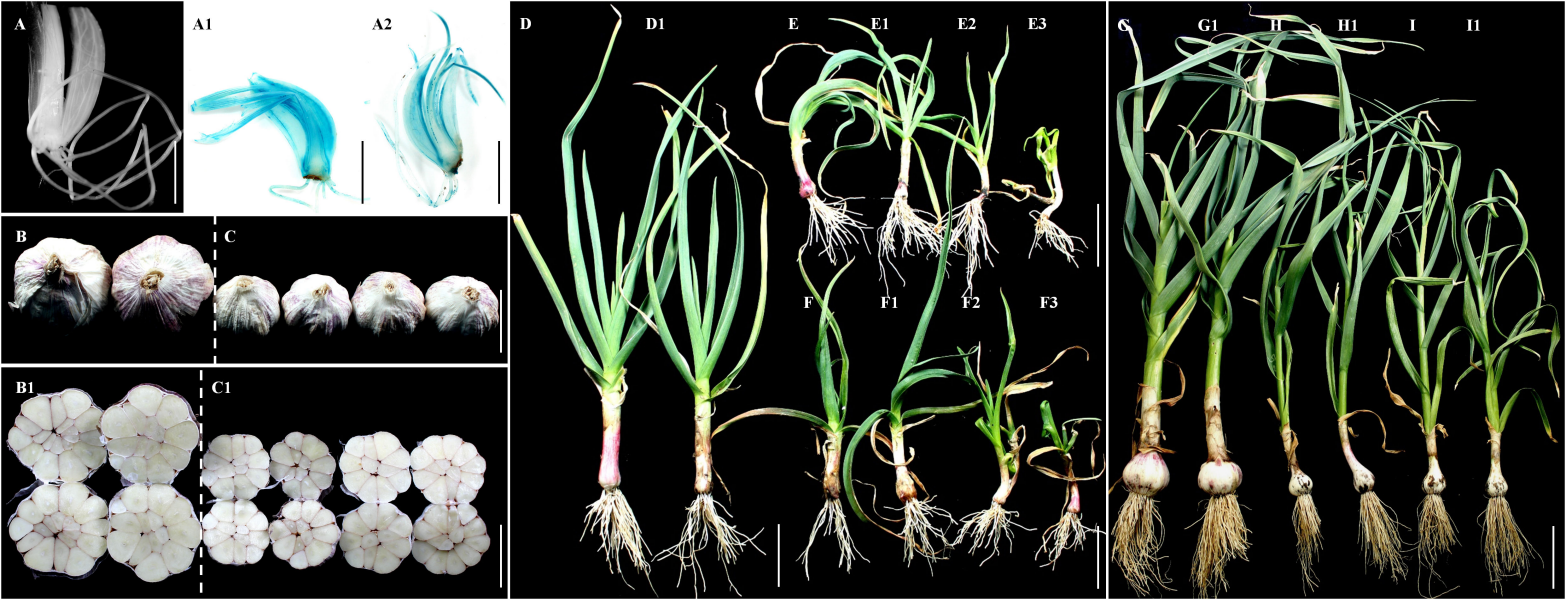
GUS histochemical staining analysis and phenotypic variations of *RcLEC1-B*-OE garlic lines. **(A-A2)** GUS histochemical assay of freehand sections from regenerated garlic sprouts. **(A)** Wild-type garlic sprout. **(A1)** Positive pBI121 garlic sprout regenerated *via* the FELB pathway. **(A2)** Positive pBI121 garlic sprout regenerated *via* the RTB pathway. **(B)** Wild-type garlic bulbs. **(B1)** Transverse section of wild-type garlic bulbs **(C)** Garlic bulbs of *RcLEC1-B*-OE lines. **(C1)** Transverse section of *RcLEC1-B*-OE garlic bulbs. **(D)** Regenerated wild-type plant *via* the FELB pathway at 150 days. **(D1)** Regenerated wild-type plant *via* the RTB pathway at 150 days. (**(E)-E3**) *RcLEC1-B*-OE lines regenerated *via* the FELB pathway at 150 days. (**(F)-F3**) *RcLEC1-B*-OE lines regenerated *via* the RTB pathway at 150 days. (**(G)**, **G1**) Regenerated wild-type plants with normal growth stature and flat leaves at 210 d. **(G)** FELB-regenerated line. **(G1)** RTB-regenerated line. (**(H)**, **H1**) *RcLEC1-B*-OE plants regenerated *via* the FELB pathway at 210 d, exhibiting dwarf phenotype as well as wrinkled and curved leaves. (**(I)**, **I1**) *RcLEC1-B*-OE plants regenerated via the RTB pathway at 210 d, showing dwarf stature and distorted, curved leaf morphology. Scale bars **(A-A2)** = 1 cm, scale bars **(B, B1, C, C1)** = 4 cm, scale bars **(D, D1, E-E3, F-F3)** = 6 cm, scale bars **(G, G1, H, H1, I, I1)** = 10 cm.

In this study, root segment explants were directly infected with *Agrobacterium*, followed by induction of FELB and RTB structures on the infected explants; regenerated plants were subsequently obtained from these FELBs and RTBs, respectively. Freehand sections of regenerated garlic sprouts from pBI121 empty vector-transformed plants-regenerated *via* the FELB and RTB pathways, respectively-exhibited blue staining in GUS histochemical assays (**Figure 6A1, A2**).

Statistical analysis of genetic transformation efficiency showed that the highest transformation efficiencies of the FELB and RTB pathways from garlic root explants reached approximately 62% and 46%, respectively. Compared to wild-type plants, *RcLEC1-B*-OE resulted in slightly dwarfed plants with flat, curved leaves that were obviously thickened, accompanied by the occurrence of double-tillering plants (**Figure 6E3, F2, F3**). All euphyllas exhibited folding due to defective cuticles (**Figure 6E–E3, F–F3**). Meanwhile, *RcLEC1-B*-OE also showed a slowed growth rate.

### 3.5 *P. chrysogenum* and *P. porri* growth and host response

**Figure 7:**
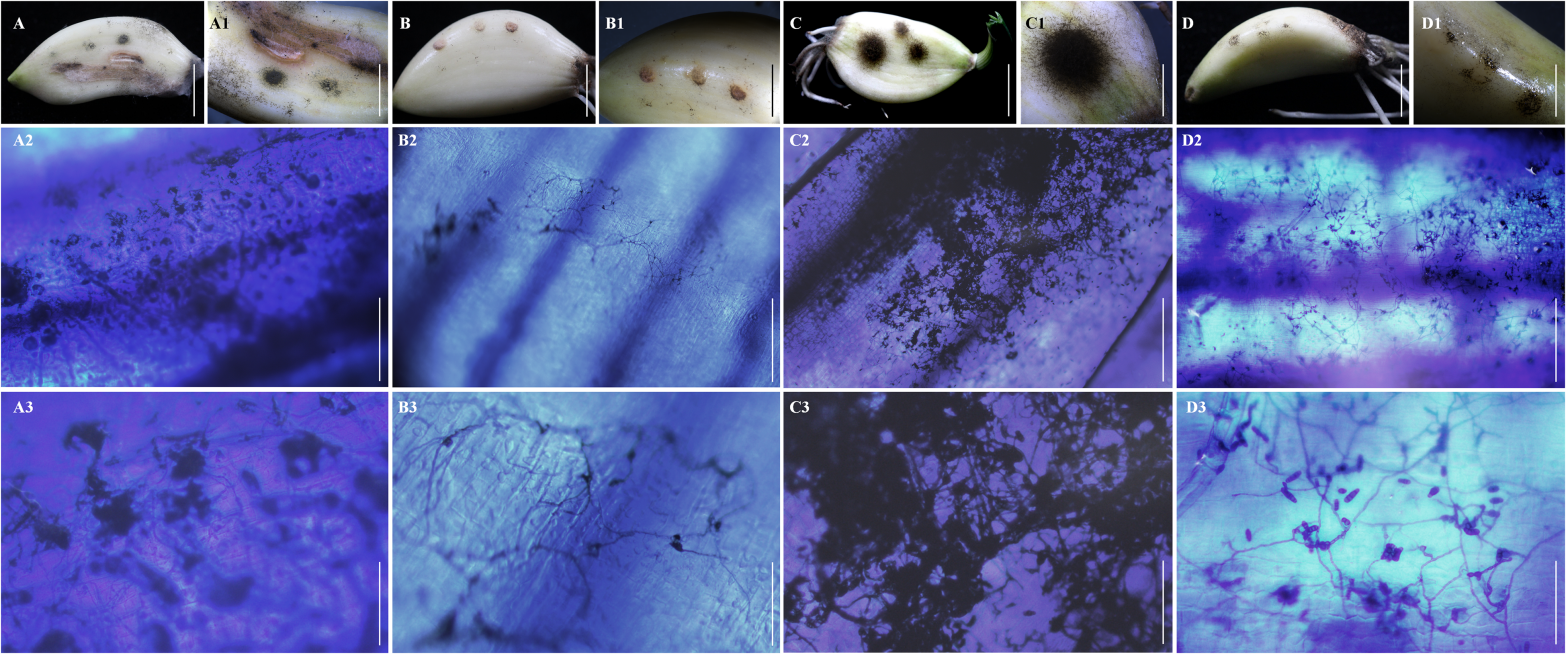
Growth characteristics of *P. chrysogenum* and *P. porri* and corresponding host responses in garlic. **(A)** Wild-type garlic cloves inoculated with *P. chrysogenum* for 17 days. **(A1)** Magnified view of **(A)**. **(A2)** Micrographs showing *P. chrysogenum* colonization on wild-type garlic cloves at 17 days post-inoculation. **(A3)** Magnified view of **(A2)**. **(B)** pCAMBIA2300*-TkTCS*-OE garlic cloves inoculated with *P. chrysogenum* for 17 days. **(B1)** Magnified view of **(B)**. **(B2)** Micrographs showing *P. chrysogenum* colonization on pCAMBIA2300*-TkTCS*-OE garlic cloves at 17 days post-inoculation. **(B3)** Magnified view of **(B2)**. **(C)** Wild-type garlic cloves inoculated with *P. porri* for 28 days. **(C1)** Magnified view of **(C)**. **(C2)** Micrographs showing *P. porri* colonization on wild-type garlic cloves at 28 days post-inoculation. **(C3)** Magnified view of **(C2)**. **(D)** pCAMBIA2300*-TkTCS*-OE garlic cloves inoculated with *P. porri* for 28 days. **(D1)** Magnified view of **(D)**. **(D2)** Micrographs showing *P. porri* colonization on pCAMBIA2300*-TkTCS*-OE garlic cloves at 28 days post-inoculation. **(D3)** Magnified view of **(D2)**. Scale bars **(A-D)** = 1 cm, scale bars (A1, B1, C1, D1) = 0.5 cm, scale bars **(A2, B2, C2, D2)** = 25 μm, scale bars **(A3, B3, C3, D3)** = 150 μm.

Microscopic observations revealed that upon infection with *P. chrysogenum* and *P. porri*, wild-type garlic plants exhibited rapid and extensive pathogen-induced cell necrosis, followed by rapid fungal growth and conidiophore formation within a short period (**Figure 7A–A3 and C–C3**).

In contrast, the infection progression was significantly impeded in *TkTCS*-OE plants. Unlike the susceptible wild-type, most cells invaded by the primary haustoria of *P. chrysogenum* and *P. porri* in *TkTCS*-OE plants retained their viability and did not undergo rapid cell death, thereby effectively inhibiting the further growth and colonization of *P. chrysogenum* (**Figure 7B2 and B3**) and *P. porri* (**Figure 7D2 and D3**). Notably, the proportion of dead cells among those attacked by fungal haustoria was drastically lower in *TkTCS*-OE plants. Statistical analysis of over 500 haustorium-invaded cells per microscopic sample showed that the average percentages of necrotic cells in wild-type vs. *TkTCS*-OE plants were approximately 49% vs. 12% for *P. chrysogenum* infections and 75% vs. 29% for *P. porri* infections, respectively.

Compared to susceptible wild-type plants, *TkTCS*-OE plants maintained cell viability in the majority of haustorium-invaded cells. Because Penicillium species are typically necrotrophic pathogens that feed on dead host tissues, this suppression of cell death in *TkTCS*-OE plants effectively restricted nutrient availability for the fungi, thereby preventing continued fungal growth and conidiophore formation. These results clearly indicate that *TkTCS*-OE plants exhibit enhanced disease resistance by suppressing pathogen-induced cell necrosis, in sharp contrast to the extensive cell death and successful fungal colonization observed in susceptible wild-type plants.

## 4 Discussion

Plant hormone analogues are commonly used as inducing factors for somatic embryogenesis (Yan et al., 2025; Zhou et al., 2025), and this is also the case for the induction of FELB and RTB in garlic. However, our study revealed a notable finding, the combined application of NAA and 2,4-D yielded an induction effect far superior to that of either hormone used alone, this mutual reinforcing phenomenon has not been previously reported in FELB and RTB induction research. This trend was consistent whether evaluating the induction of translucent mucilaginous callus or FELB. Specifically, the induction efficiency of translucent mucilaginous callus reached 98.433% (**Table 4**), while that of FELB embryos reached 96.333% (**Table 7**). For the induction of rhizoids and RTB, compared with the induction effect under full-strength sucrose concentration, the average number of rhizoids induced per explant nearly doubled under half-strength sucrose, which aligns with findings in microspore embryogenesis (Barinova et al., 2004; Touraev et al., 1996; Raina and Irfan, 1998), pollen embryogenesis (Ogawa et al., 1994), and FELB induction (Xu et al., 2016). Notably, we observed that explants (including rhizoids) could not be maintained on half-strength sucrose media for an extended period, typically no longer than 150 days. This observation may be associated with insufficient carbon source supply affecting biomass viability. Our results show that applying half-strength sucrose starvation stress for an appropriate duration promotes rhizoid induction in garlic. The underlying mechanisms driving these beneficial effects in garlic, as well as the applicability of this approach to other plant species, warrant further investigation.

Light conditions also exerted a significant influence on both stages of somatic embryogenesis in garlic. Specifically, the first stage-rhizoid induction-required explants to be maintained in darkness, while the second stage-RTB induction from rhizoids-utilized alternating dark/light cycles. Our tests on the effects of light on rhizoid and RTB induction revealed a clear contrast: complete darkness yielded the highest rhizoid induction efficiency, whereas high light intensity proved most effective for RTB induction. This observation demonstrates distinct regulatory effects of light on rhizoid and RTB induction in garlic, highlighting that optimizing light conditions is important for successful somatic embryogenesis in this species.

In garlic, rhizoids are induced from root segment, while RTBs originate from the cortical cells of these rhizoids. Notably, this study is the first to observe the early emergence of FCDZs during the rhizoid stage. In our previous research, RTBs were identified in the Cucurbitaceae family, including species such as *T. kirilowii* (Xu et al., 2015), *Cucumis sativus*, *Lagenaria siceraria*, *Cucurbita pepo*, *Momordica charantia*, and *Luffa cylindrica*. Recent preliminary unpublished data indicate that RTBs can be induced in multiple other plant families by modifying induction conditions, these families span a diverse range, including Solanaceae, Fabaceae, Linaceae, Campanulaceae, Crassulaceae, Gesneriaceae, Compositae, Rutaceae, Aquifoliaceae, Gramineae, Orchidaceae, Liliaceae, Iridaceae and Amaryllidaceae. We also observed that the explants used for rhizoid and RTB induction may vary across different plant families, which leads to differences in induction frequencies, with a notable distinction between monocotyledonous and dicotyledonous plants.

Among the seven selected explants of garlic, root segments emerged as the optimal explant for inducing translucent mucilaginous callus, FELB, rhizoids, and RTB. Despite variations in induction protocols-including distinct requirements for light/dark conditions and types or concentrations of hormone analogues-root segments consistently outperformed other explants. Analysis of the developmental processes of FELB and RTB revealed a shared sequence of stages: pro-embryo, globular-shaped embryo, and torpedo-shaped transitional embryo. Structurally, these embryos are similar to the previously reported FELB in *S. nigrum* (Xu et al., 2014) and *R. indica* (Xu et al., 2016), as well as the RTB in *T. kirilowii* (Xu et al., 2015). Their developmental trajectory did not differ significantly from traditional somatic embryogenesis processes that distinguish monocots from dicots. This study further confirmed that both mature FELB and RTB structures are organic aggregates of multiple embryos-i.e., each structure contains numerous embryos-which is reflected in the number of regenerated seedlings; this observation is consistent with previous findings in dicotyledonous plants (Xu et al., 2014, Xu et al., 2015, Xu et al., 2016).

Plant genetic transformation primarily relies on direct regeneration methods and *Agrobacterium*-mediated transgenic operations based on these approaches. In most cases, indirect transformation in plants is conducted following the formation of embryonic structures (e.g., embryonic callus and somatic embryos). However, this conventional approach carries the risk of transformation failure, attributed to the fragility of these embryonic structures and the occurrence of chimerism. In the present study, we adopted an EPST strategy: direct *Agrobacterium* infection of root segment explants, followed by induction of FELBs and RTBs from the infected explants, and subsequent plant regeneration. This approach significantly improved the infection, regeneration, and transformation efficiencies in garlic. Specifically, the genetic transformation efficiencies of the EPST method *via* the FELB and RTB pathways reached approximately 62% and 46%, respectively. Using the original root segments (which induce FELBs and RTBs) as recipient materials for genetic transformation offers two key advantages: first, these explants can induce the formation of multiple embryo aggregates, thereby generating a larger number of transformed lines; second, since FELBs and RTBs originate from single cells, this avoids the potential risk of chimerism that may occur when using somatic embryos as explants. Notably, this study is the first to successfully establish a transgenic system *via* a somatic embryogenesis-mediated method in an Amaryllidaceae plant, highlighting the novelty and applicability of the EPST strategy in monocotyledonous species within this family.

GUS histochemical assay results demonstrated that both FELB- and RTB-mediated induction methods successfully enabled the transformation of exogenous genes, with the *GUS* reporter gene expressing normally in recipient plants. To further validate the stability and reliability of the established transgenic system, we employed *RcLEC1-B* as an additional reporter gene for subsequent functional identification. Previous studies have documented multiple biological functions of *RcLEC1-B*, including impacts on plant height, tillering, fertility, fruit-setting rate, and yield in Arabidopsis and *R. sceleratus*, with notable effects such as cuticle developmental defects leading to leaf curling and wrinkling (Xu et al., 2019a, Xu et al., 2019b). Consistent with these prior findings, both 150-day-old and 210-day-old *RcLEC1-B*-OE garlic plants exhibited significant leaf curling and reduced plant height (**Figure 6H**, H1, I, I1); particularly, their developing and mature bulbs were markedly smaller than those of wild-type garlic plants (**Figure 6**B1, C1). Taken together, these results collectively confirm the successful establishment of an efficient regeneration and genetic transformation system based on the FELB and RTB pathways in garlic, providing a reliable technical platform for future genetic improvement and functional genomic research in this crop.

In the present study, we evaluated the agronomic traits (e.g., disease resistance) of *TkTCS*-OE garlic plants. Our results demonstrated that the yield and quality of *TkTCS*-OE garlic were not adversely affected by the overexpression of *TkTCS*. Through comprehensive analyses of mycelial growth and host defense responses, we confirmed that *TkTCS*-OE garlic exhibited significantly enhanced resistance to *P. chrysogenum* and *P. porri*. These findings show strong consistency with functional transgenic studies of trichosanthin in other economically vital monocot staples. In rice, stable *TkTCS*-OE markedly inhibits rice blast fungus (*Pyricularia oryzae*) proliferation, with no negative impacts on plant growth or grain yield recorded in positive transgenic lines (Ming et al., 2000; Yuan et al., 2002). Particle-mediated *TkTCS* transformation in wheat also produces virus-resistant transgenic progeny with uncompromised agronomic performance (Xu et al, 2001). Broad reviews of plant RIPs confirm a conserved mode of action across monocot lineages: moderate heterologous *TkTCS* expression selectively impairs pathogen ribosomal translation while avoiding toxic interference with host plant protein synthesis, triggering amplified endogenous defense cascades against fungal invaders (Zhu et al., 2018; Kumar et al., 2023). Nevertheless, our garlic research presents distinct breakthroughs relative to cereal monocot *TkTCS* trials. The vast majority of rice and wheat *TkTCS* assays address field foliar or root pathogens, while this work targets destructive postharvest bulb rot-the primary production constraint limiting garlic bulb storage lifespan and market value. Additionally, garlic possesses notoriously low regeneration and transformation efficiency compared to easily engineered cereals; our stably expressing *TkTCS* transgenic garlic germplasm provides a rare validated technical system for molecular improvement of recalcitrant *Allium* bulb monocots, a research frontier minimally explored in existing RIP transgenic literature. These findings not only highlight the potential of Trichosanthin as a candidate gene for improving garlic disease resistance but also provide valuable genetic resources for molecular design breeding strategies aimed at enhancing the postharvest preservation of garlic bulbs and cloves. This is particularly relevant for garlic production, as postharvest decay caused by *Penicillium* and *Phytophthora* species remains a major constraint to yield and quality retention.

## 5 Conclusion

In brief, we developed two high-efficiency regeneration and transformation systems for garlic *via* FELB and RTB induction. Root segments served as superior explants; NAA combined with dark incubation induced translucent mucilaginous callus and FELBs, while sucrose semistarvation, Elevated concentrations of NAA and TDZ combined with intense light exert synergistic effects to stimulate massive formation of rhizoids and RTBs, which facilitates somatic embryogenesis. Phenotypic observation of *RcLEC1-B*-OE lines and GUS staining verified a stable transformation system, overcoming garlic’s historically low transformation efficiency. Disease resistance tests on *TkTCS*-OE bulbs supply germplasm for disease resistance and storage improvement, alongside a pathogen interaction assay framework. This work advances monocot somatic embryogenesis theory and delivers a robust technical platform for garlic and Amaryllidaceae crop gene function analysis and molecular breeding.

## Data Availability

The original contributions presented in the study are included in the article/supplementary material. Further inquiries can be directed to the corresponding authors.
